# How to assess chemically defined media and feeds from 9 suppliers on CHO cells producing mAb

**DOI:** 10.1186/1753-6561-7-S6-P100

**Published:** 2013-12-04

**Authors:** Aurore Polès-Lahille, Margaux Paillet, Aurélie Da Silva, Nora Kadi, Eric Basque, Flavien Thuet, David Balbuena, Sébastien Ribault

**Affiliations:** 1 Merck Biodevelopment, Martillac, France, 33650

## Introduction

Mammalian cell culture medium development has widely evolved in recent years. The use of hydrolysates as serum replacement has led to process variability due to lot-to-lot variations. The undefined composition of these media could also increase the process optimization timelines, sometimes with limited impact on process performances. With the reduction of process development activities for preclinical and Phase I studies, medium and feed platforms raised. The objective of the media was to ensure cell growth only in order to go as fast as possible to production bioreactors while the feeds were responsible for productivity and production length. Either companies spent several months if not years to develop their own generic medium and feed platforms or they used commercial ones, sometimes under licenses. The medium and feed platform assessment also started earlier in the product development process. Clone screening was performed more and more in fed-batch conditions rather than batch ones. Thus screening tools, scale-down models of bioreactors, with lower and lower working volumes were designed. Another cell culture process evolution was the development of new expression systems without any selection agents. In order to assess our screening scale-down model, between 20 to 35 chemically defined platforms from 9 suppliers were screened with 3 CHO host cell lines/expression systems.

## Methods

The following protocol was followed for 3 different CHO cell lines producing mAb:

- CHO host cell 1 - expression system n°1 : mAb I

- CHO host cell 1 - expression system n°2 : mAb II

- CHO host cell 2 - expression system n°3 : mAb III

Each medium was prepared, supplemented according to cell requirements, 0.2 μm PVDF filtrated and stored into at least 2 separated bottles. A sterility test was performed on each bottle before use. Each cell line was thawed and amplified during at least one week in its usual medium. Then the cells were adapted to each medium for at least 8 passages in either 125 mL shake flasks or 50 mL spin tubes in duplicate. Each media was preheated at 37°C before use and one bottle was used per duplicate in order to reduce contamination risk. After cell adaptation, fed-batch platform assessment was performed in 50 mL spin tubes at 37°C with a seeding density around 0.25 * 10^6 ^viable cells/mL. Every 2 to 3 days, samples were taken to measure pH, pO_2_, pCO_2_, viable cell density, viability, glucose and lactate levels. The feeding strategy applied was the same for each cell line and agreed with each supplier. The cultures were stopped when the viability was below 60% or after 16-17 days.

## Results

The objective of cell culture media is to sustain cell growth in order to quickly seed the production bioreactor. Here are the doubling times measured on the 3 cell lines (Table 1).

**Table 1:** Doubling time of each cell line in each medium after adaptation.

	mAb I	mAb II	mAb III
Cellvento™ CHO-200 medium	20 h	32 h	17 h

Supplier A medium 1	23 h	35 h	23 h

Supplier A medium 2	20 h	63 h	22 h

Supplier A medium 3	20 h	24 h	18 h

Supplier B medium 1	21 h	20 h	18 h

Supplier B medium 2	24 h	66 h	24 h

Supplier C medium 1	26 h	18 h	20 h

Supplier C medium 2	22 h	18 h	18 h

Supplier D medium 1	21 h	19 h	18 h

Supplier E medium 1	26 h	35 h	28 h

Supplier E medium 2	23 h	20 h	19 h

Supplier F medium 1	21 h	26 h	17 h

Supplier G medium 1	21 h	20 h	18 h

Supplier G medium 2			19 h

Supplier G medium 3			21 h

Supplier G medium 4			19 h

Supplier H medium 1			21 h

Supplier H medium 2			21 h

**Figure 1:**
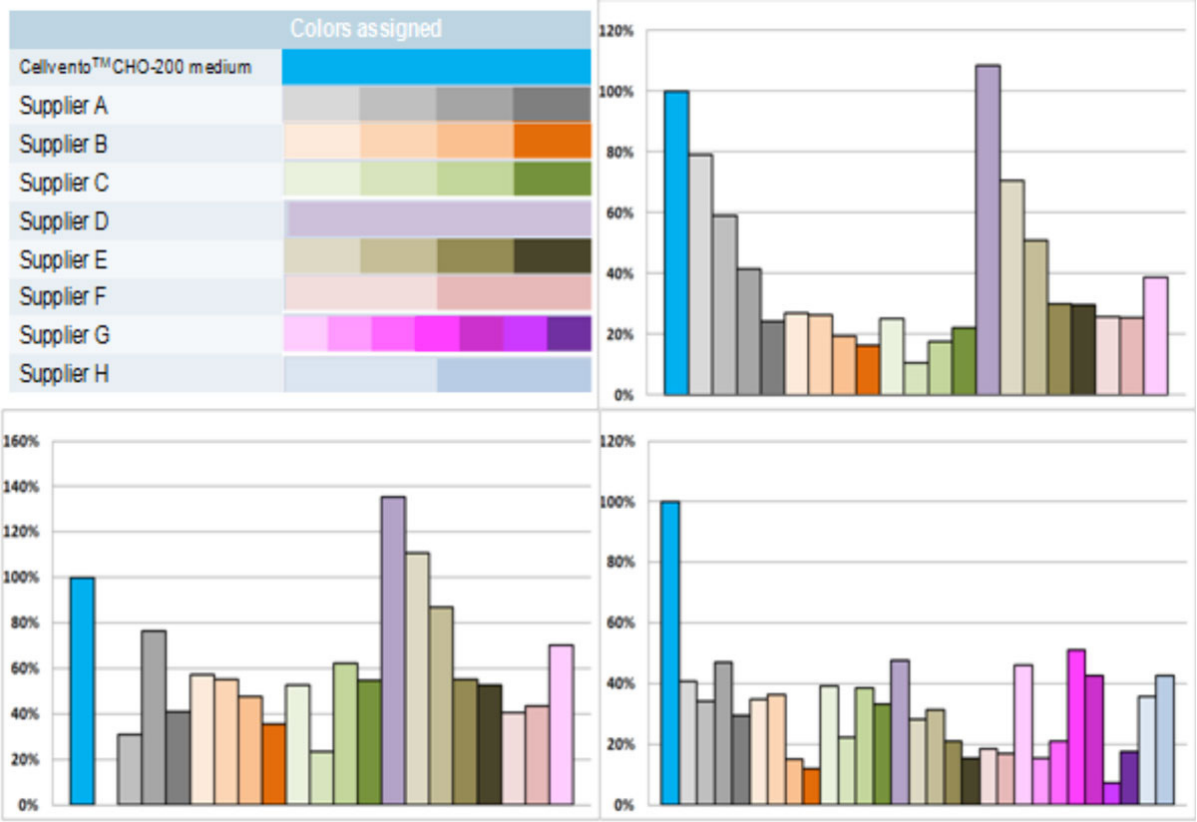
**Colors assigned to each supplier**. Final mAb I (top right side), mAb II (bottom left side) and mab III (bottom right side) titers for all platforms compared to Cellvento ™ CHO-200 medium.

Despite having the same host cell, cell growth was different between mAb I and mAb II. The expression system could have a significant impact on cell growth behavior. In order to separate the different platform results, a color was assigned to each supplier and platform assessed (Figure 1).

Depending on the CHO host cell and the expression system, each platform had different performances. Some platforms seemed to be more robust than others in terms of final titer. The lactate metabolism was also compared between the different platforms. Most of the platforms had a maximum lactate concentration measured around 1 - 1.5 g/L. Some platforms went above 2 g/L of lactate, which could be difficult to scale-up in bioreactors. The practical aspect was also studied as it can facilitate the implementation and the tech transfer. Some platforms assessed had 2 feeds added everyday while others only had 1 feed added 3 times. Molecule quality was also compared between platforms in terms of High Molecule Weight and cIEF.

## Conclusions

We have implemented a strong protocol for medium and feed screening with up to 70 spin tubes manipulated in parallel. More than 3000 sterile manipulations were performed under a laminar flow without any contaminations. These experiments allow us to define robust platforms in terms of cell growth, productivity and metabolism on different CHO host cell lines and expression systems.

